# Characteristics of unipolar depression in psychiatric inpatients

**DOI:** 10.1192/j.eurpsy.2021.906

**Published:** 2021-08-13

**Authors:** M. Kacem, S. Khouadja, S. Brahim, R. Boukhchina, L. Zarrouk

**Affiliations:** 1 Department Of Psychiatry, University hospital of mahdia, chebba, Tunisia; 2 Psychiatry, University Hospital Of Mahdia, Mahdia, Tunisia; 3 Department Of Psychiatry, University Hospital of Mahdia, chebba, Tunisia; 4 Psychiatry, University Hospital of Mahdia, Tunisia., chebba, Tunisia; 5 Department Of Psychiatry, University Hospital Of Mahdia, Tunisia., Psychiatry, Mahdia, Tunisia

**Keywords:** inpatients, unipolar, Depression

## Abstract

**Introduction:**

Unipolar depression is daily encountered in psychiatry.

**Objectives:**

To describe the socio-demographic and clinical characteristics of patients with unipolar depression.

**Methods:**

This is a cross-sectional, descriptive study carried out at the psychiatric department of the University Hospital of Mahdia. We have included patients with unipolar depression. The data were collected from patients’ medical files using a pre-established 37-item questionnaire.

**Results:**

We have collected 53 patients. The mean age was 44 years. The majority of patients were female (56.6%) and unemployed (70%). 47.2% of patients were married. 72% of patients had a low socio-economic level. They were smokers in 45.3% of cases. Alcohol consumption was found in 24.5% of cases. A family history of mood disorder and suicide or attempted suicide were present in 7% and 13.2% of the cases respectively. 7% of the patients had a history of a postpartum thymic episode. The mean number of depressive episodes was 2.5. Personal history of suicide attempts was found in 40% of cases. The mean age of the first thymic episode was 35 years. At the psychiatric examination, psychomotor retardation was present in 64% of cases, anxiety distress in 58.5% of cases, psychotic, melancholic and atypical characteristics in 30%, 13.2% and 5.7% of cases respectively. 81% of patients were treated with anxiolytic drugs in combination with an antidepressant. Antipsychotic treatment was combined in 45% of cases and electro-convulsive therapy in 9.4% of cases.

**Conclusions:**

Our patients presented predictive criteria of bipolarity. Therefore, vigilance is necessary in their medical management.

